# Genome-wide analysis of the NAC transcription factor family in Tartary buckwheat (*Fagopyrum tataricum*)

**DOI:** 10.1186/s12864-019-5500-0

**Published:** 2019-02-06

**Authors:** Moyang Liu, Zhaotang Ma, Wenjun Sun, Li Huang, Qi Wu, Zizhong Tang, Tongliang Bu, Chenglei Li, Hui Chen

**Affiliations:** College of Life Science, Sichuan Agricultural University, Ya’an, China

**Keywords:** Tartary buckwheat, *NAC* family, Genome-wide, Fruit development, Expression profile, Phylogenetics

## Abstract

**Background:**

The *NAC* (*NAM*, *ATAF1/2*, and *CUC2*) transcription factor family represents a group of large plant-specific transcriptional regulators, participating in plant development and response to external stress. However, there is no comprehensive study on the *NAC* genes of Tartary buckwheat (*Fagopyrum tataricum*), a large group of extensively cultivated medicinal and edible plants. The recently published Tartary buckwheat genome permits us to explore all the *FtNAC* genes on a genome-wide basis.

**Results:**

In the present study, 80 *NAC* (*FtNAC*) genes of Tartary buckwheat were obtained and named uniformly according to their distribution on chromosomes. Phylogenetic analysis of NAC proteins in both Tartary buckwheat and *Arabidopsis* showed that the FtNAC proteins are widely distributed in 15 subgroups with one subgroup unclassified. Gene structure analysis found that multitudinous *FtNAC* genes contained three exons, indicating that the structural diversity in Tartary buckwheat *NAC* genes is relatively low. Some duplication genes of *FtNAC* have a conserved structure that was different from others, indicating that these genes may have a variety of functions. By observing gene expression, we found that *FtNAC* genes showed abundant differences in expression levels in various tissues and at different stages of fruit development.

**Conclusions:**

In this research, 80 *NAC* genes were identified in Tartary buckwheat, and their phylogenetic relationships, gene structures, duplication, global expression and potential roles in Tartary buckwheat development were studied. Comprehensive analysis will be useful for a follow-up study of functional characteristics of *FtNAC* genes and for the development of high-quality Tartary buckwheat varieties.

**Electronic supplementary material:**

The online version of this article (10.1186/s12864-019-5500-0) contains supplementary material, which is available to authorized users.

## Background

Transcription factors (TFs) play an important role in controlling multitudinous vital growth and development processes such as signal transduction, cellular morphogenesis, and response to environmental stressors during the growth and development of plants [1, 2]. TFs activate or inhibit the transcription rate of target genes by binding to specific cis-acting promoter elements to regulate gene expression [3, 4]. The NAC domain protein family is a larger plant-specific TF family. The family name is based on the three proteins: no apical meristem (NAM), ATAF1–2, and cup-shaped cotyledon (CUC), which all possess a similar DNA-binding domain [5, 6]. Typically, the structure of NAC proteins can be divided into conserved N-terminal DNA-binding domains and highly dispersed C-terminal transcriptional regulatory regions [7, 8]. NAC domains comprise five subdomains, A through E, located at the N-terminal, and usually contain approximately 150 amino acid residues, which are related to DNA binding, dimer formation and localization [9–11].

Since the *NAC* gene was first found to influence pattern formation in embryos and flowers of petunias [5], numerous studies have shown that the NAC protein is important for plant growth and environmental adaptation. [11, 12]. *NAC* genes affect many important biological processes. In *Arabidopsis*, *NAP* is involved in floral morphogenesis [13]. At*NAC*1 and At*NAC*2 are involved in the development of lateral roots [14, 15]. *CUC1* is the second gene indispensable for the formation of cup-shaped cotyledons [16]. *ATAF1/2* and *CUC2* participate in cell division [17]. *RD26* and A*NAC*036 are involved in hormone signal pathways [18, 19]. *NAM-B1* is involved in the deposition of nutrients in wheat grains [20, 21]. *OsNAC5* may participate in nutrient substance remobilization from green tissues to seeds [22].

*NAC* genes are also important regulators of plant abiotic stress response [11]. In *Arabidopsis*, overexpressing *ANAC019/055*/*072* enhanced tolerance to drought by stress-induced gene expression [23]. In rice, the overexpression of *OsNAC6/SNAC2* enhanced seedling tolerance to drought, salt, and cold stress [24, 25]. In *Arabidopsis*, the heteroexpression of active membrane-bound GmNTL1/GmNLT11 proteins was found to result in improved tolerance of abiotic stressors [26]. In *Capsicum*, silencing *CaNAC2* increased the sensitivity of seedlings to cold stress [27]. In Tartary buckwheat, *FtNAC4/6/7/8/9* was involved in responses to drought, high salt, cold, SA and JA stress [28]. In addition, *NAC* transcription factors play an important role in secondary cell wall (SCW) biosynthesis in plants [29, 30]. In *Arabidopsis*, the dominant repression of *VND6*/*7* (vascular-related NAC domain) causes an inhibition of secondary wall thickening in vessels [31]. *NST1* and *NST3* (NAC secondary wall thickening promoting factor) regulated secondary wall biosynthesis, including production of xylary and interfascicular fibers and pod shattering, in a functionally redundant manner [32–34]. *AtXND1* (xylem NAC domain 1) negatively regulated terminal secondary wall biosynthesis [35]. In *Medicago sativa*, an *MtNST1* gene mutant exhibited a lack of lignification in interfascicular fibers [36]. In cotton, transgenic experiments indicate that *GhFSN1* positively regulates SCW thickness [37].

Tartary buckwheat is a widely cultivated dual-purpose medicinal and food grain crop that contains abundant proteins with a well-balanced composition of essential amino acids, and its content of amino acids is higher than that of primary grain crops. Tartary buckwheat is a rich source of vitamins B1, B2 and B6, dietary fiber and a variety of minerals, such as niacin, magnesium, manganese and phosphorus (adopted from USDA Food Composition Databases: https://ndb.nal.usda.gov/ndb/). Importantly, Tartary buckwheat is a rich source of beneficial phytochemicals. Flavonoids, especially rutin, have anti-fatigue properties and anti-inflammatory activity and can be used to treat microangiopathy [38–41]. Although *NAC* genes affect cell division and expansion [17], lateral root development [14, 15], maintenance of the shoot apical meristem [5], flower formation [13] and secondary cell wall biosynthesis [29, 30], there are still many problems to be solved. The *NAC* gene family has been widely studied in many species, such as *Arabidopsis thaliana* [7], rice [42], wheat [43], soybean [44], maize [45], potato [46], cassava [47], Chinese cabbage [48], pepper [49], melon [50] and apple [51]. However, there is no systematic study of the *NAC* family in Tartary buckwheat. Due to the fact that NAC genes are crucial to many developmental processes and responses to abiotic stress, it is of great significance to comprehensively study the *NAC* family of Tartary buckwheat. The recent completion of Tartary buckwheat genome sequencing allowed us to study the *NAC* gene family of Tartary buckwheat at the genome level [52]. To date, only the *ARF* and *MADS* gene families have been systematically analyzed in Tartary buckwheat [53, 54]. In this article, we report the gene structure, chromosomal location, duplication, global expression and potential developmental roles of Tartary buckwheat *NAC* genes. In addition, a homologous analysis between *NAC* genes in *Arabidopsis thaliana*, beet, soybean, tomato, sunflower, rice, grape and Tartary buckwheat was also conducted. The expression of *FtNAC* genes during plant development, especially fruit development, was comprehensively analyzed. This study is helpful as a follow-up study of the functional characteristics of *FtNAC* genes in the development of Tartary buckwheat.

## Results

### Identification of *FtNAC* genes in Tartary buckwheat

Eighty *NAC* genes were identified by the two BLAST methods, and the redundant forms of the same gene were removed simultaneously. In this study, *FtNACs* were named *FtNAC 1* to *FtNAC 80* according to their chromosome location (Additional file 1: Table S1).

We introduced the basic information of 80 *NAC* genes in detail, including isoelectric point (PI), molecular weight (MW) and coding sequence length (CDS). Of the 80 FtNAC proteins, the smallest protein was *FtNAC 37* with 91 amino acids, and the largest protein was *FtNAC 24* (621 aa) (Additional file 1: Table S1). The MWs of the proteins ranged from 10.59 to 69.92 KDa, and the pIs ranged from 4.36 (*FtNAC 7*) to 11.46 (*FtNAC 58*). From the location information, we can see that 69 FtNACs were located in the nucleus, 4 FtNACs were located in the cytoplasm, 3 FtNACs were located in the chloroplast, 3 FtNACs were located in the mitochondria, and 1 FtNAC was located in the plasma membrane (Additional file 1: Table S1).

### Phylogenetic analysis and classification of *FtNAC* genes

**Fig. 1:**
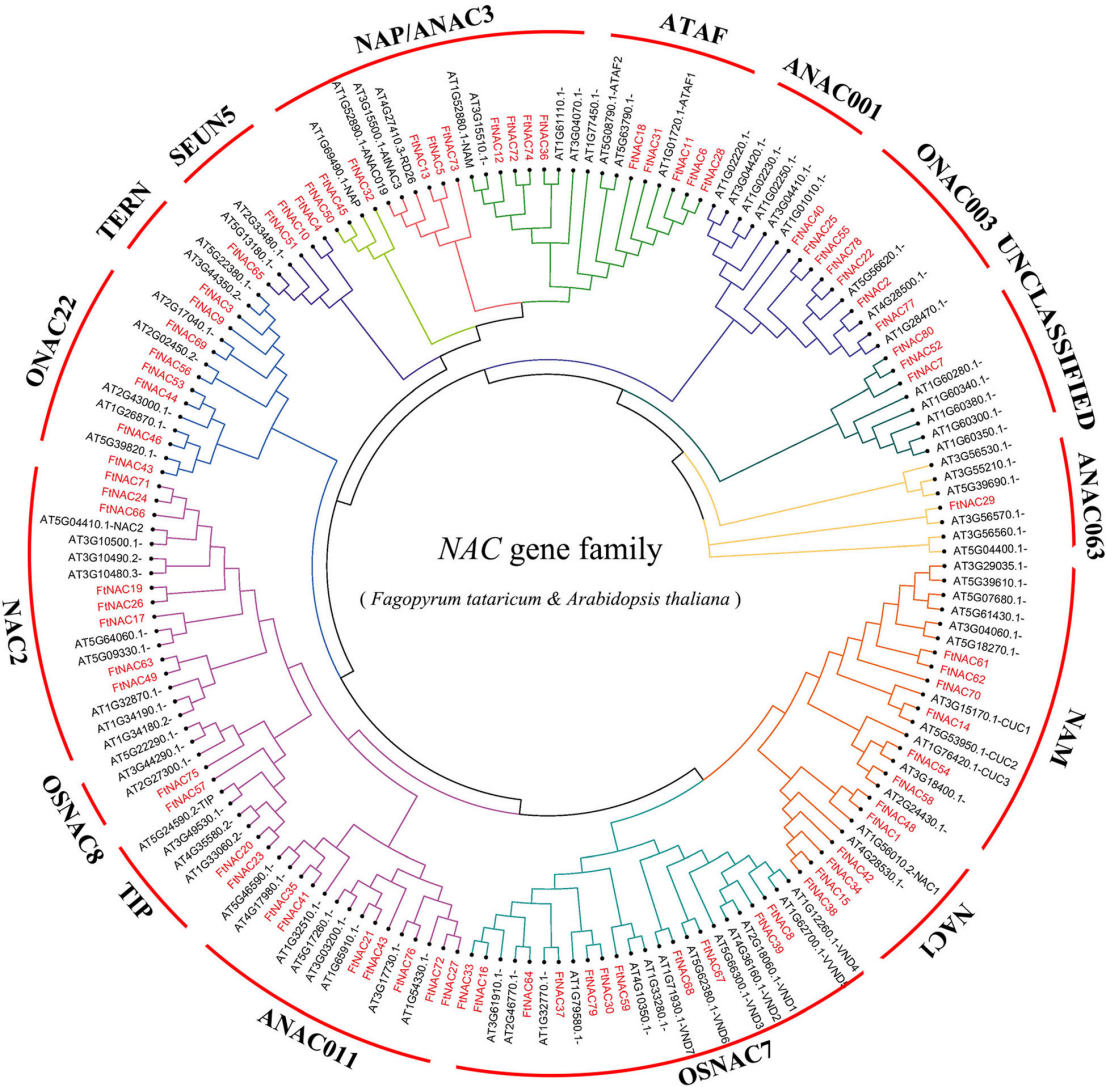
Unrooted phylogenetic tree representing relationships among the NAC proteins of Tartary buckwheat and *Arabidopsis*. The tree divided the FtNAC proteins into 16 subgroups represented by different colored clusters within the tree. A phylogenetic tree was constructed from the NAC protein sequence of Tartary buckwheat and *Arabidopsis thaliana*. The phylogenetic trees were derived using the neighbor-joining (NJ) method in Geneious R11. The parameters used included a Blosum62 cost matrix, the Jukes-Cantor model, and global alignment with free end gaps and bootstrap value of 1000

**Fig. 2:**
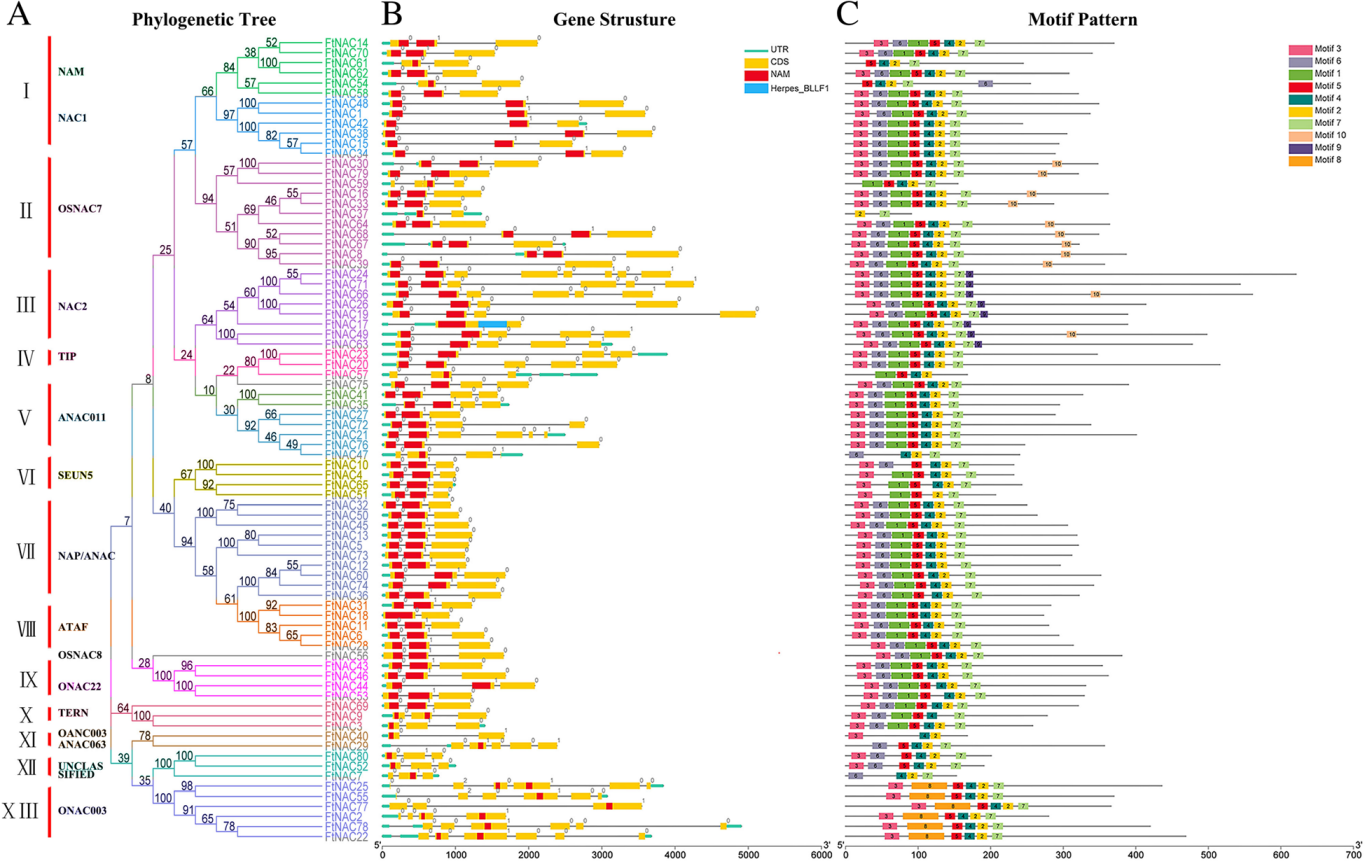
Phylogenetic relationships, gene structure and architecture of conserved protein motifs in *NAC* genes from Tartary buckwheat. **a** The phylogenetic tree was constructed based on the full-length sequences of Tartary buckwheat NAC proteins using Geneious R11 software. **b** Exon-intron structure of Tartary buckwheat *NAC* genes. Green boxes indicate untranslated 5′- and 3′-regions; yellow boxes indicate exons; black lines indicate introns. The NAM domains are highlighted by red boxes. The number indicates the phases of the corresponding introns. **c** The motif composition of Tartary buckwheat NAC proteins. The motifs, numbered 1–10, are displayed in different colored boxes. The sequence information for each motif is provided in Additional file 2. The length of the protein can be estimated using the scale at the bottom

We used amino acid sequences of FtNAC and AtNAC proteins to construct an unrooted phylogenetic tree to explore their evolutionary relationship. According to their homology with NAC proteins in *Arabidopsis*, 80 *FtNAC* genes were divided into 14 subgroups (Fig. 1). In our analysis, we found that the NAC proteins in Tartary buckwheat had representatives from the ANAC063, NAM, NAC1, OsNAC7, ANAC011, TIP, OsNAC8, NAC2, ONAC22, TERN, SENU5, NAP/ANAC3, ATAF, and ONAC003 subgroups. However, in Tartary buckwheat, no NAC members from the ANAC001 subgroup were identified. The subgroups OSNAC8 and ANAC063 each contained only one FtNAC protein, whereas the subgroup OSNAC7 contained the greatest number (11) of FtNAC proteins. The ATNAC3 and NAP members represented a single clade (Figs. 1 and 2a). In an earlier analysis, these two classes were reported to be sister taxa, suggestive of their coevolution [47, 55]. These results indicate that the functions of *FtNAC* genes in Tartary buckwheat are diverse, which is consistent with the reports in *Arabidopsis* and rice.

### Gene structure and motif composition of the *FtNAC* gene family

A phylogenetic tree was constructed from 80 amino acid sequences of FtNAC, and the gene family was divided into thirteen subgroups, named I-XIII, according to bootstrap values and motif composition (Fig. 2a). The largest, subgroup I, had 12 members, whereas the subgroups V and X had only two members. Two *NAC* genes, *FtNAC75* and *FtNAC56,* could not be assigned to any subgroup because they had low bootstrap values. To analyze the structure of *FtNAC* genes, we compared the genomic DNA sequences to analyze the composition of introns and exons (Fig. 2b). The coding sequences of the entire *FtNAC* family were interrupted by introns, with the number of exons ranging from 1 to 8 (Fig. 2b). *FtNAC24* had 7 introns, whereas *FtNAC17* contained no introns in the open reading frame (ORF). The gene structure is relatively conserved within the *NAC* gene family; among its members, the majority of genes have 3 exons (Fig. 2b). In general, the closest members from the same subgroups had a similar exon/intron structure with regard to intron number, intron phase, and exon length.

To further study the characteristic regions of FtNAC proteins, the motifs of 80 FtNAC proteins were analyzed online using MEME. Ten conserved motifs were predicted, and the specific amino acid sequences of each motif were also provided (Fig. 2c, Additional file 2: Table S2). We found that most of the closely related members in the phylogenetic tree showed common motifs with the same alignment and position, which indicates that the members of NAC that clustered in the same subgroup may have similar biological functions. Most of the FtNAC proteins contained motif 3 (subdomain A), motif 6 (subdomain B), motif 1 and motif 5 (subdomain C), motif 4 and motif 2 (subdomain D), and motif 7 (subdomain E) in succession. Notably, some conserved motifs in specific subgroups were identified. All of the FtNAC proteins in subgroup XIII lacked motif 1 and motif 6 and contained motif 8, and all of the members of subgroup III contained motif 9. Additionally, all of the FtNAC proteins in subgroup II contained motif 10, except for *FtNAC37* and *FtNAC74*, which contained only 2 motifs. This pattern suggests that the specific motifs may be related to specific functions of different subgroups. The motif composition and gene structure of the members from each subgroup obtained from phylogenetic analysis were similar, which indicates that the classification was more reliable.

### Evolutionary analysis of *FtNAC* genes and several different species

**Fig. 3:**
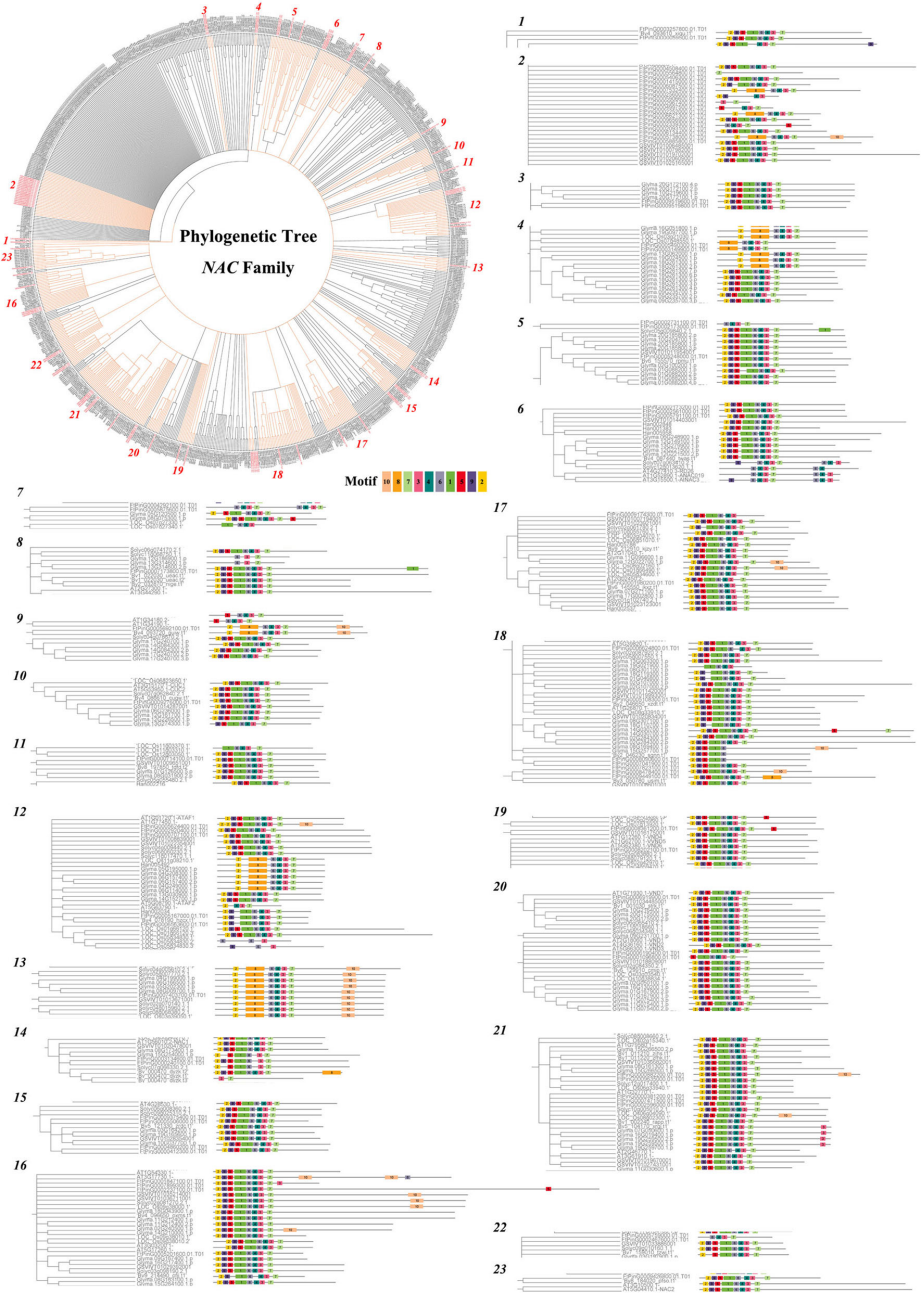
Phylogenetic relationships and motif compositions of NAC proteins from 8 different plant species. Left panel: an unrooted phylogenetic tree constructed using the neighbor-joining method in Geneious R11. Right panel: distribution of conserved motifs in NAC proteins. The different colored boxes represent different motifs and their positions in each NAC protein sequence

In comparison with the existing *NAC* genes of Tartary buckwheat, we also studied the evolutionary diversification of *NAC* genes. A phylogenetic tree was constructed containing the NAC protein sequences from one monocotyledonous plant (rice) and seven dicotyledonous plants (*Arabidopsis thaliana*, Tartary buckwheat, beet, soybean, tomato, sunflower and grape) using Geneious R11. All NAC proteins were divided into twenty-three subclades within the phylogenetic tree. However, a number of sequence relationships were not well defined (Fig. 3).

Using MEME, ten different conserved motifs were found, among which motif 2 and motif 9, motif 5, motif 1 and motif 6, motif 4 and motif 3, and motif 7 encoded NAC subdomains A-E, respectively. (Fig. 3). Notably, all of the NACs without motifs 9, 5 and 1 had motif 8 in the N-terminal but not in the C-terminal (Fig. 3). Additionally, some NACs contained at least one of the motifs 1, 3, 4, 5, 6, 7, or 9 in the C-terminal and N-terminal simultaneously (Fig. 3). The majority of NAC proteins in the same clade, particularly the most closely related proteins, typically shared common motifs (for example, *FtNAC49* and *Glyma.08G009700.1.p*), indicating a similar potential function between these NAC proteins.

### Chromosomal location and synteny analysis of *FtNAC* genes

**Fig. 4:**
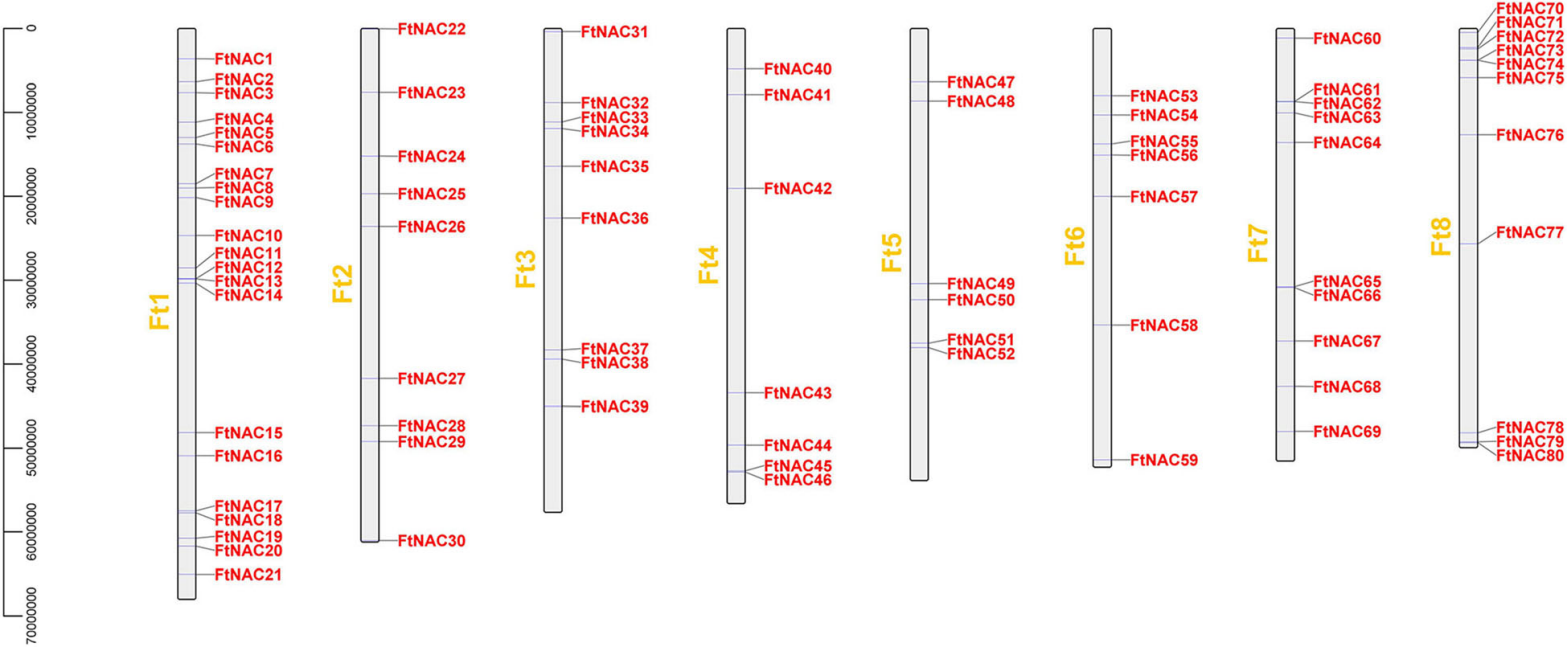
Distribution of *FtNAC* genes among 8 chromosomes. Vertical bars represent the chromosomes of Tartary buckwheat. The chromosome number is to the left of each chromosome. The scale on the left represents chromosome length

**Fig. 5:**
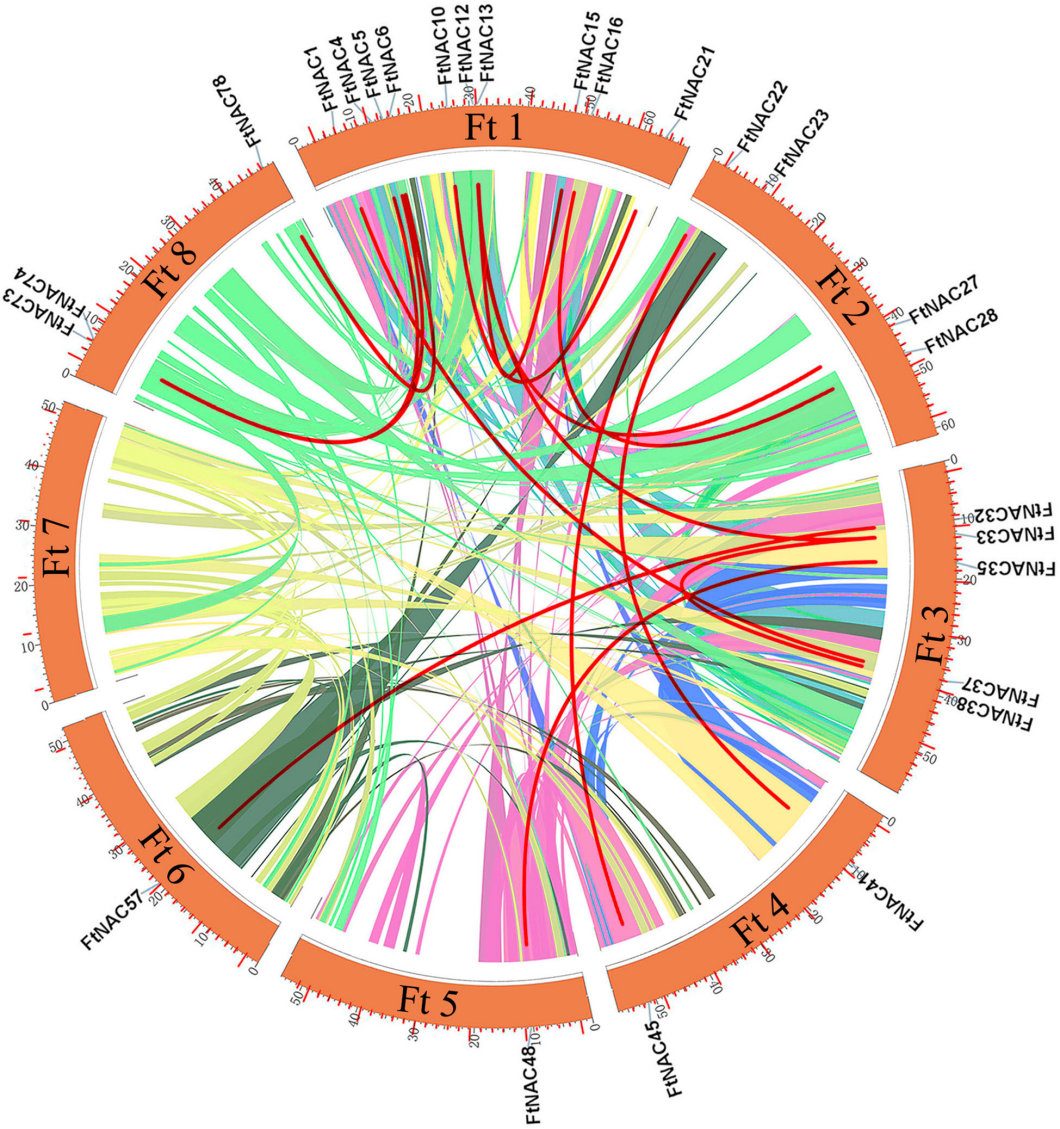
Schematic representations of the interchromosomal relationships of Tartary buckwheat *NAC* genes. Colored lines indicate all synteny blocks in the Tartary buckwheat genome, and the red lines indicate duplicated *NAC* gene pairs. The chromosome number is indicated below each chromosome

A total of 80 *FtNAC* genes were unevenly distributed on 8 chromosomes of Tartary buckwheat (Fig. 4). The largest, chromosome 1, had the most *FtNAC* genes (21, ~ 25.93%), followed by the smallest, chromosome 8 (11, ~ 13.58%) and chromosome 7 (10, ~ 12.35%), whereas there were only six genes in chromosome 5 (~ 1.31%) (Fig. 4). To identify the duplication events in *FtNAC* genes, a collinearity analysis was performed using MCScanX software. Fourteen pairs of fragment duplication genes were found in 80 *FtNAC* genes (Fig. 5). Fragment duplication genes were the most common on chromosome 1, followed by chromosome 3, whereas there were no fragment duplication gene pairs on chromosome 7 (Fig. 5).

**Fig. 6:**
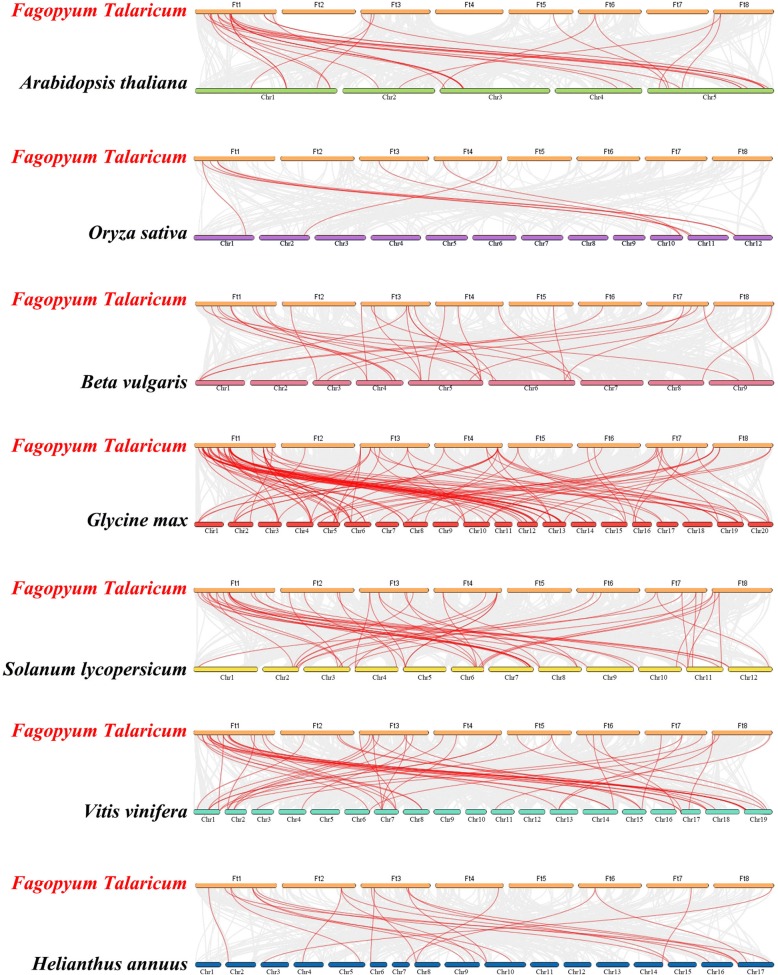
Synteny analysis of *NAC* genes between Tartary buckwheat and seven representative plant species. Gray lines in the background indicate the collinear blocks within the Tartary buckwheat and other plant genomes, whereas the red lines highlight the syntenic *NAC* gene pairs

To further explore the evolutionary relationship of the Tartary buckwheat NAC family, we constructed a phylogenetic tree consisting of six dicotyledonous plants (*Arabidopsis*, beet, tomato, soybean, sunflower and grape) and a monocotyledonous plant (rice) (Fig. 6). The order of genome size was as follows: sunflower (3.6 Gb) [56], soybean (1.025 Gb) [57], tomato (828.349 Mb) [58], beet (714–758 Mb) [59], grape (490 Mb) [60], rice (466 Mb) [61] and *Arabidopsis thaliana* (125 Mb) [62]. In total, 43 *FtNAC* genes displayed a syntenic relationship with grape, followed by soybean (40), tomato (40), beet (29), *Arabidopsis* (20), sunflower (15) and rice (6). The number of homologous pairs in the other seven species (soybean, tomato, *Arabidopsis thaliana*, grape, beet, sunflower and rice) were 73, 36, 28, 24, 21, 17 and 6, respectively (Additional file 3: Table S3). Tartary buckwheat chromosome 1 had the most homologous gene pairs with other species. In addition, the number of gene pairs between Tartary buckwheat and other species was not related to the genome size of the other species.

Some *FtNAC* genes were formed in at least six syntenic gene pairs, particularly those of Tartary buckwheat and soybean, such as *FtNAC3* and *FtNAC46*, which may play a vital role in the evolution of the *NAC* gene family. Tartary buckwheat and rice shared less than 10 colinear gene pairs, which may be associated with the phylogenetic relationship between these species.

### Expression patterns of *FtNAC* genes in different plant tissues

**Fig. 7:**
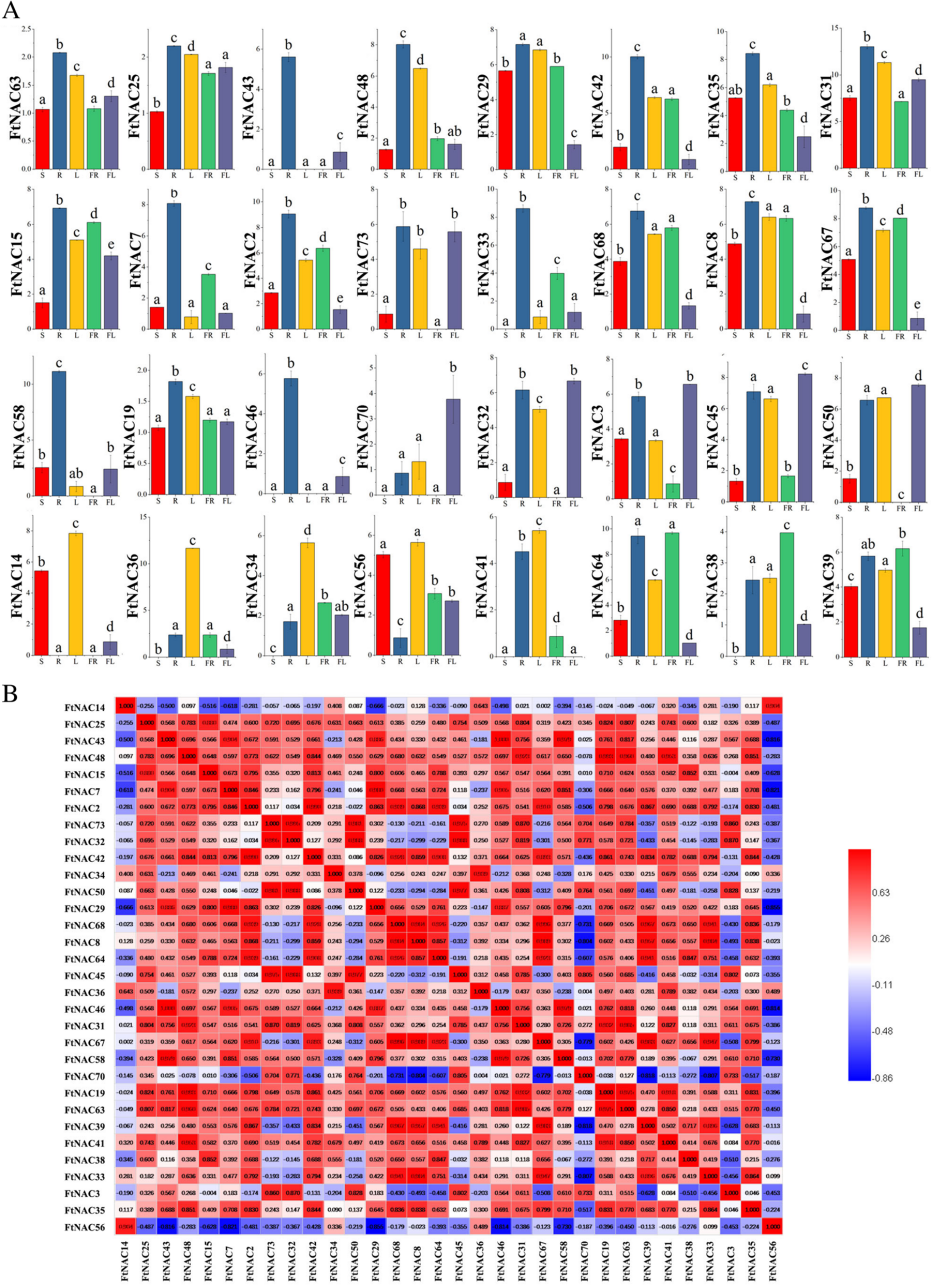
Tissue-specific gene expression of 32 Tartary buckwheat *NAC* genes and the correlation between the gene expression of FtNACs. **a** The expression patterns of 32 Tartary buckwheat *NAC* genes in flower, leaf, root, stem and fruit tissues were examined using a qPCR assay. **b** The correlation between the gene expression of FtNACs. Red: positively correlated; Blue: negatively correlated. Error bars were obtained from three measurements. Small letter(s) above the bars indicate significant differences (α = 0.05, LSD) among the treatments

To explore the physiological role of the *FtNAC* genes, we randomly selected 32 genes distributed among the branches (Fig. 2, Additional file 4: Table S4) and performed qPCR. The expression levels of 32 *FtNAC* genes in stem, root, leaf, fruit and flower tissues were evaluated (Fig. 7a). As shown in Fig. 7, the various expression patterns of each gene in different tissues indicated that *FtNAC* genes perform different functions in Tartary buckwheat.

Three *FtNAC* genes (*FtNAC43, FtNAC46* and *FtNAC58*) were more highly expressed in roots than in other organs. (Fig. 7a). *FtNAC70* was highly expressed in flower tissue, and *FtNAC36* was highly expressed in leaf tissue (Fig. 7a). FtNAC14 was highly expressed in both leaf and stem tissue (Fig. 7a). *FtNAC31* was highly expressed in all organs of Tartary buckwheat and may play crucial roles in adaptation to the environment during plant growth and development. In addition, the expression patterns of some *FtNAC* genes in the same subgroup were similar in Tartary buckwheat, such as four genes in subgroup VII *(FtNAC73, FtNAC32, FtNAC50* and *FtNAC45*) and three genes in subgroup II *(FtNAC68, FtNAC67* and *FtNAC8*) (Figs. 2 and 7a). Furthermore, *FtNAC32* and *FtNAC45* were duplicated *FtNAC* members. However, some duplicated *FtNAC* genes (*FtNAC15* and *FtNAC38, FtNAC35* and *FtNAC41*, etc.) in the same subgroup showed diffe rent expression patterns (Figs. 2, 5 and 7a), indicating that the functions of these genes may have changed during the evolution of Tartary buckwheat. In general, understanding the expression patterns of *FtNAC* genes in different tissues can lay a foundation for identifying functional genes in Tartary buckwheat.

We also studied the correlation of expression patterns for 32 *FtNAC* genes in Tartary buckwheat (Fig. 7b). Most of the *FtNAC* genes were positively correlated, and all of the *FtNAC* genes that were significantly correlated (*FtNAC43* and *FtNAC58/FtNAC46/ FtNAC33/FtNAC7*, *FtNAC14* and *FtNAC56*, *FtNAC42* and *FtNAC68/FtNAC64/FtNAC67/FtNAC2*, etc.) were found to be positively correlated (Fig. 7b).

### Differential expression of *FtNAC* genes during fruit development in Tartary buckwheat

**Fig. 8:**
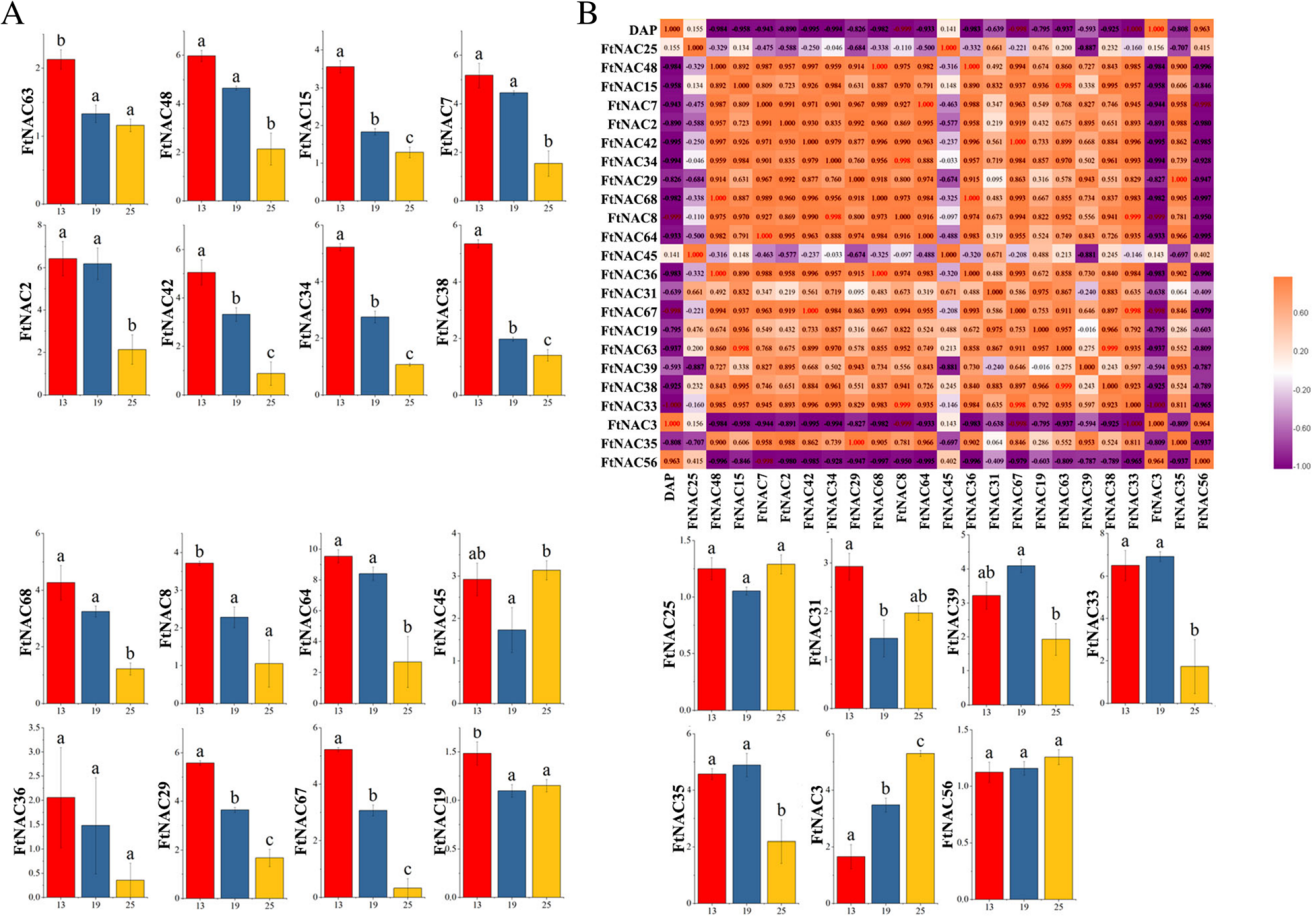
Gene expression of 23 Tartary buckwheat *NAC* genes during fruit development and the correlation between the gene expression of FtNACs. **a** The expression patterns of 23 Tartary buckwheat *NAC* genes at three fruit development stages were examined using a qPCR assay. **b** The correlation between the gene expression of FtNACs. Orange: positively correlated; Purple: negatively correlated. Error bars were obtained from three measurements. Small letter(s) above the bars indicate significant differences (α = 0.05, LSD) among the treatments

*NAC* transcription factors have been studied in many plants in relation to plant development and biological stress, but only a few studies have evaluated their role in fruit development. Tartary buckwheat is a nutritional and medicinal crop, and as with other cereal crops, the fruit of the Tartary buckwheat is the most important part and of the greatest concern. The expression levels of 23 *FtNAC* genes in three fruit development stages were determined by qPCR to explore the function of these genes. (Fig. 8a). The expression of three *FtNAC* genes (*FtNAC42*, *FtNAC34* and *FtNAC15*) showed a consistent decreasing trend across the three fruit development stages, whereas *FtNAC3* showed an increasing trend (Fig. 8a). The expression of *FtNAC45* initially decreased and then increased across the three fruit development stages. *FtNAC56* was expressed at a similar level at all three stages.

We also studied the correlation of expression patterns for 23 *FtNAC* genes at three fruit development stages in Tartary buckwheat (Fig. 8b). Based on the correlation results, we observed that most *FtNAC* genes were positively correlated and that the *FtNAC* genes were significantly correlated (*FtNAC29* and *FtNAC67/FtNAC8*, *FtNAC6* and *FtNAC38/ FtNAC15*, etc.) were found to be positively correlated (Fig. 8b).

## Discussion

### *FtNAC* gene identification and evolutionary analysis in Tartary buckwheat

The NAC domain protein family is a larger plant-specific TF family. In recent years, 117 proteins have been identified in *Arabidopsis thaliana* [7], 151 in rice [42], 167 in cassava [47], 104 in pepper [49], and 180 in apple [51], but there are few studies on the NAC family in Tartary buckwheat. However, in previous studies, eight *FtNAC* genes were cloned and found to respond to at least one treatment, among which *FtNAC4(FtNAC5)* and *FtNAC7(FtNAC31)* showed extremely significant responses to salt, drought, ABA, and SA treatments [28]. In the current study, we identified 80 members of the *FtNAC* family (Fig. 1), which were designated *FtNAC1* to *FtNAC80* on the basis of their chromosomal locations (Fig. 4, Additional file 1: Table S1). Clearly, the *NAC* gene family in Tartary buckwheat is by far the smallest compared with that in other plant species. This size difference can be attributed to the genome of Tartary buckwheat containing fewer *NAC* genes than other plants, probably because more duplication events have occurred in other species after differentiation from their earliest ancestors. The duplication of genes can amplify the number of genes; for example, four large duplication events have occurred in the *Arabidopsis* genomes [63, 64]. The ρ genome duplication (WGD) event is inferred to have occurred after different lineages, while the σWGD event occurred in all Poales [65]. Hence, the low number of *FtNAC* genes may be due to the absence of a ρWGD event during the evolution of Tartary buckwheat. The genome size of each species was very different (such as Tartary buckwheat, 489.3 Mb [52]; *Arabidopsis thaliana*, 125 Mb [62]; rice, 466 Mb [61]; soybean, 1.025 Gb [57]), indicating that the *NAC* gene family was stable in different species during evolution. Although the length, MW and pI of *NAC* genes vary greatly, the structure of the genes were relatively conservative; among these *FtNACs*, the majority of genes have 3 exons (Fig. 3b), while the number of exons ranged from 1 to 8 [43]. The gene structure of *NAC* genes in Tartary buckwheat was similar to that of *NAC* genes in rice and cassava [42, 47]. A collinearity analysis of the data in this study showed that there were 14 pairs of fragment replication genes without tandem replication events (Fig. 5). Therefore, chromosome fragment replication events drive the evolution of *FtNAC* genes, which greatly facilitates the expansion of the *NAC* gene family in plants with smaller genomes. The duplicated *FtNAC* genes may have functional redundancy. Among the 14 duplicated gene pairs, *FtNAC32* and *FtNAC45* have a similar, consistent pattern of expression. In the current theory, duplicated genes may pass through 4 selection modes [66], which may result in differences in expression patterns. There is evidence of this from the expression patterns of *FtNAC35* and *FtNAC41*. *FtNAC35* had the highest expression level in roots, followed by stems, while *FtNAC41* had no expression in stems and the highest expression in leaves. In our study, the *NAC* genes in the phylogenetic tree of related species were relatively similar, and many branches contained the *NAC* genes from dicotyledons and monocotyledons, which indicates that the diversity of *NAC* genes appeared before the differentiation of monocotyledons and dicotyledons (Fig. 3) [67]. Collinearity analysis showed that there were only 10 pairs of homologous genes in Tartary buckwheat and rice, whereas more orthologous gene pairs were found in six of the dicotyledonous leaves, including those of soybean and tomato (Fig. 6). Consistent with the phylogenetic relationship between Tartary buckwheat and the other seven plants, this analysis suggests that the *NAC* genes in dicotyledonous leaves are more homologous and consistent.

### Functional analysis of special structures of *FtNAC* genes in Tartary buckwheat

The domains and motifs of transcription factors are often related to protein interaction, transcriptional activity and DNA binding [68]. The conserved motifs in the N-terminus of the *NAC* genes have highly conserved DNA-binding abilities (Fig. 2c), which indicates that these motifs are very important for the function of *NAC* genes [7]. Motif analysis showed that most of the *NAC* genes of Tartary buckwheat contained motif 1 to motif 7 (Fig. 2c). However, among the *FtNAC* members in subgroup XIII, motif 8 (DEFIPTLDGEDGICYTHPEKLPGVTKDGLARHFFHRPSKAYTTGTRKRRK) replaced motifs 1 (part of subdomain C, binds to DNA) and 6 (subdomain B, has distinctive functions) (Fig. 2c), which might confer a distinctive function to this subgroup [7, 11]. In an evolutionary analysis of *NAC* genes in several different plants, it was also found that motif 8 (EFIPTJEGEBGICYTHPEKLPGVTKDGLSRHFFHRPSKAYTTGTRKRRKI) replaced motifs 5 (part of subdomain A, promotes functional dimerization), 9 (subdomain B) and 1 (part of subdomain C), although they were not all present together (Fig. 3). The genes of subgroup XIII cluster with the ONAC003 subgroup containing *Arabidopsis AT4G28500*, which has been found to function in the formation of secondary cell wall fibers [69]. *FtNAC25* and *FtNAC2*, the genes of subgroup XIII, were expressed in all tissues. We speculate that most of the genes in subgroup XIII have similar expression patterns due to their common conserved motifs and thus play a role in secondary wall formation.

### Tartary buckwheat *NAC* genes may play an important role in plant development, particularly in fruits

Valuable insights regarding the roles of *FtNAC* genes involved in specific Tartary buckwheat physiological processes were obtained from the different expression patterns of *FtNAC* genes. The biological function of new proteins is typically assigned through alignment with homologous sequences in *Arabidopsis*. Several studies have assigned a wider range of functions to NAC proteins using this approach, such as temperature stress in Chinese cabbage [48] and dehydration stress in soybean [44]. In a multispecies phylogenetic tree, genes in one branch often have the same function. Therefore, the function of NAC in Tartary buckwheat was predicted by clustering with *Arabidopsis* NAC protein sequences in the phylogenetic tree. Gene expression is a basic form of evidence for gene function. qPCR analysis revealed that *FtNAC70* may be involved in the development of flowers. *FtNAC43*, *FtNAC46* and *FtNAC58* may play a role in the roots because they exhibit a higher expression level in roots. *FtPinG0009426800.01* was the most highly expressed in leaves and may play a role in leaf development. The expression pattern of *FtNAC15* was different from that of *FtNAC38*, although the two genes are duplicated genes within the same subgroup. The NAM groups may regulate cell division and leaf development [16, 17, 70–73]. The NAM subgroup gene *FtNAC14* is most highly expressed in the leaf followed by the stem and is orthologous with *AT5G53950.1(CUC2)*, indicating that *FtNAC14* may function in leaf development through expression in both the leaf and stem (Fig. 7a). The multispecies evolutionary analysis showed that they fall in the same subgroup and have the same motif composition (Fig. 3). The proteins of a given subgroup contain the same motif, indicating that the *NAC* genes of the same subgroup may perform similar functions [7]. The expression of *FtNAC56* was significantly positively correlated with the expression of *FtNAC14* (Fig. 7b). The overexpression of homologous genes *AT2G02450.1 (LOV1)* in switchgrass alters the leaf angle, cell wall composition, and flowering time [56]. *FtNAC56* was also most highly expressed in the leaf followed by the stem, suggesting that these two genes have synergistic functions in leaves and stems. Although they belong to different subgroups, they have the same motif composition (Figs. 2 and 7a). Thus, *FtNAM* may participate in leaf development. These results suggest that *FtNAC* genes are extensively involved in the root, stem and leaf development of Tartary buckwheat.

Among the 32 *FtNAC* genes, most were expressed in fruits, although 8 *FtNAC* genes were not expressed (Fig. 7a). Therefore, we explored the expression pattern of 23 *FtNAC* genes at three developmental stages of Tartary buckwheat fruit. Fruit development is an essential process of cell division, differentiation and expansion, which determines fruit quality. In Tartary buckwheat, the most noticeable changes were fruit size and texture, which were related to the activity of different genes [74, 75]. In previous studies, the development of Tartary buckwheat fruit was divided into five stages [76]. We selected three representative stages (13DAP, 19DAP and 25DAP) corresponding to the initial filling stage, milk stage and initial maturity stage to study the role of *FtNAC* genes in fruit development. *FtNAC45*, which belongs to the NAP and AtNAC3 subgroups, initially decreased and then increased during the three fruit development stages and may play a role in the fruit development of Tartary buckwheat. *FtNAC45* and *FtNAC31* showed similar expression patterns at 13 DAP and 19 DAP but exhibited differences at 25 DAP. The overexpression of *SlNAC1* alters fruit pigmentation and softening [77], whereas the suppression of tomato *SlNAC1* delays fruit ripening [78]. *ATAF2* is orthologous to *SlNAC1* [79] and is involved in auxin biosynthesis [80], which influences the fruit size of Tartary buckwheat [74]. There was a positive correlation between auxin concentrations and cell division or final seed size in a comparison of two kinds of Tartary buckwheat from 7 to 19 DAP [74]. The size of the fruit is mainly determined by the early development of the embryo, not by the later stage [81, 82]. *FtNAC31* is orthologous to *AT5G08790.1* (*ATAF2*) (Fig. 3) and is highly expressed at 13 DAP and may function in the early stages fruit development in Tartary buckwheat. Both *FtNAC67* and *FtNAC68* are highly expressed at 13 DAP and 19 DAP. These two genes cluster to OSNAC7. Genes in this subgroup have been reported many times to function in secondary wall biosynthesis [29–34, 36, 37, 83]. This result suggests that the *FtOSNAC7* genes function in the secondary wall biosynthesis of Tartary buckwheat fruit, with the development from 13 DAP~ 19 DAP, Tartary buckwheat fruit gradually developed completely [76]. According to the expression patterns of *FtNAC* genes, it can be speculated that these genes may be involved in similar regulatory networks, but the specific function of *FtNAC* genes in Tartary buckwheat fruit needs to be further verified.

## Conclusion

In our study, a comprehensive analysis including gene structure, chromosomal location, sequence homology synteny and expression patterns of the *NAC* gene family in Tartary buckwheat was first performed. We identified a total of 80 *FtNAC* genes in Tartary buckwheat, which will provide basic information for the functional characterization of *FtNAC* genes. Furthermore, expression analysis of *FtNAC* genes in various tissue and developmental stages of fruit implied that *FtNACs* may participate in the development of Tartary buckwheat. These data are useful for further exploration of *NAC* gene-mediated physiological processes in Tartary buckwheat. This systematic analysis of the *NAC* gene in Tartary buckwheat is helpful for the follow-up study of the functional characteristics of *FtNAC* genes and the cultivation of high-quality Tartary buckwheat varieties.

## Methods

### Identification of FtNAC genes in Tartary buckwheat

We downloaded the genome of Tartary buckwheat from the TBGP (Tartary Buckwheat Genome Project; http://www.mbkbase.org/Pinku1/). The *FtNAC* genes were obtained using two BLASTP methods. First, all AtNACs were used to search possible FtNACs on the TBGP website, and further identified using BLASTP [84] (score value ≥100 and e-value ≤1e^− 10^) [53]. Second, we downloaded the hidden Markov model (HMM) file of the NAC domain (PF 02365) through Pfam (http://pfam.xfam.org/). All genes were retrieved using HMMER 3.0 (default parameters) with a cutoff of 0.01 [85]. The possible FtNACs were confirmed with PFAM and SMART programs [86, 87], and the HMMERs were used to further verify the results. Finally, all NAC gene models were used to analyze the CDS length, MW, PI and subcellular localization using tools on the ExPasy website.

### Analysis of gene structure

To explore the structure of *FtNAC* genes, ClustalW (default parameters) was used [88]. We used the GSDS (Gene Structure Display Server) analysis to analyze the constituents of the exons/introns of the *FtNAC* genes. We used MEME to analyze the motifs of FtNAC proteins, (http://meme-suite.org/tools/meme), with the parameters as follows: motif width set to 6–200 and number set to 10 [53, 89].

### Chromosomal location, gene duplication and evolutionary analysis of *FtNAC* genes

Circos were used to locate *FtNAC* genes on the chromosomes of Tartary buckwheat. We used MCScanX (default parameters) to examine duplication genes [90]. We analyzed the homology of the *NAC* gene between Tartary buckwheat and the other seven plants using Dual Synteny Plotter (https://github.com/CJ-Chen/TBtools).

### Phylogenetic analysis and classification of the *FtNAC* gene family

According to the classification of AtNAC, all the identified *FtNAC* genes were divided into different groups. NAC protein sequences (from *Arabidopsis*, rice, beet, soybean, tomato, sunflower and grape) were obtained by UniProt https://www.uniprot.org/). The NJ phylogenetic trees were constructed using Geneious R11 with Blosum62 cost matrix, the Jukes-Cantor model, global alignment with free end gaps and bootstrap value of 1000.

### Plant growth

Xiqiao is produced by physical mutagenesis and is characterized by higher rutin and crude protein contents. It has many advantages, such as a high seed setting rate, strong anti-falling ability, high 1000-grain weight and many grains per plant [94]. Xiqiao was planted in a field of the Sichuan Agricultural University (29°97’ N, 102°97′ E; elevation, 580 m), Ya’an, Sichuan, China and in a consistent growth environment for five consecutive years since 2013. The flower, fruit from three different fruit developmental stages (13, 19, and 25 DAP) [76], stem, root, and leaf from the maturity period were collected from three different Tartary buckwheat plants with identical developmental statuses. Samples were quickly frozen in liquid nitrogen after collection and stored at − 80°C before use.

### Expression analysis of *FtNAC* genes using qPCR

The selected gene expression analysis used qPCR, and primers were designed by Primer3 (Additional file 4: Table S4). We used the *FtH3* gene, which was stably expressed at each growth stage in almost all tissues, as an internal control [53]. The qPCR with SYBR Premix Ex Taq II (TaKaRa) was repeated at least three times and the data were analyzed using the 2^−(∆∆Ct)^ method [91].

The data were analyzed using Origin Pro 2018b, and the means were compared using LSD at the 0.05 and 0.01 levels of significance.

## Additional files

Additional file 1: **Table S1.** List of the 80 *FtNAC* genes identified in this study. (XLS 184 kb)

Additional file 2: **Table S2.** Analysis and distribution of conserved motifs in Tartary buckwheat NAC proteins. (XLS 31 kb)

Additional file 3: **Table S3.** One-to-one orthologous relationships between Tartary buckwheat and other plants. (XLS 88 kb)

Additional file 4: **Table S4.** The primer sequences of qRT-PCR. (XLS 32 kb)

## Abbreviations

ABA: Abscisic acid
At: *Arabidopsis thaliana*
BLASTP: Basic Local Alignment Search Tool-Protein
CDS: Coding sequence
CUC: Cup-shaped cotyledon
DAP: Days after pollination
ERFs: Ethylene response factors
Ft: *Fagopyrum tataricum*
HMM: Hidden Markov model
JA: Jasmonic acid
MW: Molecular weight
NAM: No apical meristem
NAP: NAC-like, activated by AP3/PI
NST: NAC secondary wall thickening promoting factor
PI: Isoelectric point
qPCR: Quantitative real-time PCR
SA: Salicylic acid
SCW: Secondary cell wall
TFs: Transcription factors
VND: Vascular-related NAC domain
XND: Xylem NAC Domain

## References

[1] Slobodan R, Diego RP, Ingo D, Bernd MR (2007). PlnTFDB: an integrative plant transcription factor database. Bmc Bioinformatics.

[2] He Z, Jinpu J, Liang T, Yi Z, Xiaocheng G, Ge G, Jingchu L (2011). PlantTFDB 2.0: update and improvement of the comprehensive plant transcription factor database. Nucleic Acids Res.

[3] Riechmann JL, Heard J, Martin G, Reuber L, Keddie J, Adam L, Pineda O, Ratcliffe OJ, Samaha RR, Creelman R (2000). Arabidopsis transcription factors: genome-wide comparative analysis among eukaryotes. Science.

[4] Wray G, Hahn MW, Abouheif E, Balhoff J, Pizer M, Rockman MV, Romano LA. The Evolution of Transcriptional Regulation in Eukaryotes[J]. Mol Biol Evol. 2003;20(9):1377-419. 10.1093/molbev/msg140 12777501

[5] Souer E, Houwelingen A, van Kloos D, Mol J, Koes R (1996). The no apical meristem gene of Petunia is required for pattern formation in embryos and flowers and is expressed at meristem and primordia boundaries. Cell.

[6] Aida M, Ishida T, Fukaki H, Fujisawa H, Tasaka M (1997). Genes involved in organ separation in Arabidopsis: an analysis of the cup-shaped cotyledon mutant. Plant Cell.

[7] Ooka H, Satoh K, Doi K, Nagata T, Otomo Y, Murakami K, Matsubara K, Osato N, Kawai J, Carninci P (2003). Comprehensive analysis of NAC family genes in Oryza sativa and Arabidopsis thaliana. DNA Res.

[8] Kikuchi K, Ueguchi-Tanaka M, Yoshida KT, Nagato Y, Matsusoka M, Hirano HY (2000). Molecular analysis of the NAC gene family in rice. Mol Gen Genet MGG.

[9] Jensen MK, Kjaersgaard T, Nielsen MM, Galberg P, Petersen K, O'Shea C, Skriver K (2010). The Arabidopsis thaliana NAC transcription factor family: structure-function relationships and determinants of ANAC019 stress signalling. Plant Signal Behav.

[10] Olsen NA, Ernst HA, Leggio LL, Skriver K (2005). NAC transcription factors: structurally distinct, functionally diverse. Trends Plant Sci.

[11] Puranik S, Sahu PP, Srivastava PS, Prasad M (2012). NAC proteins: regulation and role in stress tolerance. Trends Plant Sci.

[12] Wu Y, Deng Z, Lai J, Zhang Y, Yang C, Yin B, Zhao Q, Zhang L, Li Y, Yang C (2010). Dual Function of Arabidopsis ATAF1 in Abiotic and Biotic Stress Responses. 2010 Papers Collection of National Symposium on Plant Biology 2010.

[13] Sablowski RWM, Meyerowitz EM (1998). A homolog of NO APICAL MERISTEM is an immediate target of the floral homeotic genes APETALA3/PISTILLATA. Cell.

[14] Xie Q, Frugis G, Colgan D, Chua NH (2000). Arabidopsis NAC1 transduces auxin signal downstream of TIR1 to promote lateral root development. Genes Dev.

[15] He X, Mu R, Cao W, Zhang Z, Zhang J, Chen S (2010). AtNAC2, a transcription factor downstream of ethylene and auxin signaling pathways, is involved in salt stress response and lateral root development. Plant J.

[16] Takada S, Hibara K, Ishida T, Tasaka M (2001). The CUP-SHAPED COTYLEDON1 gene of Arabidopsis regulates shoot apical meristem formation. Development.

[17] Youn-Sung K, Sang-Gyu K, Jung-Eun P, Hye-Young P, Mi-Hye L, Nam-Hai C, Chung-Mo P (2006). A membrane-bound NAC transcription factor regulates cell division in Arabidopsis. Plant Cell.

[18] Fujita M, Fujita Y, Maruyama K, Seki M, Hiratsu K, Ohme-Takagi M, Tran LS, Yamaguchi-Shinozaki K, Shinozaki K (2010). A dehydration-induced NAC protein, RD26, is involved in a novel ABA-dependent stress-signaling pathway. Plant J.

[19] Hiroaki K, Taizo M, Yoshibumi K, Tamao S, Atsushi K (2010). Overexpression of the NAC transcription factor family gene ANAC036 results in a dwarf phenotype in Arabidopsis thaliana. J Plant Physiol.

[20] Cristobal U, Assaf D, Tzion F, Ann B, Jorge D (2006). A NAC gene regulating senescence improves grain protein, zinc, and iron content in wheat. Science.

[21] Waters BM, Uauy C, Dubcovsky J, Grusak MA (2009). Wheat (Triticum aestivum) NAM proteins regulate the translocation of iron, zinc, and nitrogen compounds from vegetative tissues to grain. J Exp Bot.

[22] Sperotto RA, Ricachenevsky FK, Duarte GL, Boff T, Lopes KL, Sperb ER, Grusak MA, Fett JP (2009). Identification of up-regulated genes in flag leaves during rice grain filling and characterization of OsNAC5, a new ABA-dependent transcription factor. Planta.

[23] Lam-Son Phan T, Kazuo N, Yoh S, Simpson SD, Yasunari F, Kyonoshin M, Miki F, Motoaki S, Kazuo S, Kazuko YS (2004). Isolation and functional analysis of Arabidopsis stress-inducible NAC transcription factors that bind to a drought-responsive cis-element in the early responsive to dehydration stress 1 promoter. Plant Cell.

[24] Kazuo N, Tran LSP, Dong VN, Miki F, Kyonoshin M, Daisuke T, Yusuke I, Nagao H, Kazuo S, Kazuko YS (2010). Functional analysis of a NAC-type transcription factor OsNAC6 involved in abiotic and biotic stress-responsive gene expression in rice. Plant J.

[25] Hu H, You J, Fang Y, Zhu X, Qi Z, Xiong L (2010). Erratum to: characterization of transcription factor gene SNAC2 conferring cold and salt tolerance in rice. Plant Mol Biol.

[26] Li S, Wang N, Ji D, Xue Z, Yu Y, Jiang Y, Liu J, Liu Z, Xiang F (2016). Evolutionary and functional analysis of membrane-bound NAC transcription factor genes in soybean. Plant Physiol.

[27] Guo WL, Wang SB, Chen RG, Chen BH, Du XH, Yin YX, Gong ZH, Zhang YY (2015). Characterization and expression profile of CaNAC2 pepper gene. Front Plant Sci.

[28] Deng R, Zhao H, Xiao Y, Huang Y, Yao P, Lei Y, Li C, Chen H, Wu Q (2018). Cloning, characterization, and expression analysis of eight stress-related NAC genes in Tartary buckwheat. Crop Sci.

[29] Zhou J, Zhong R, Ye ZH (2014). Arabidopsis NAC domain proteins, VND1 to VND5, are transcriptional regulators of secondary wall biosynthesis in vessels. PLoS One.

[30] Nakano Y, Yamaguchi M, Endo H, Rejab NA, Ohtani M (2015). NAC-MYB-based transcriptional regulation of secondary cell wall biosynthesis in land plants. Front Plant Sci.

[31] Minoru K, Makiko U, Nobuyuki N, Gorou H, Masatoshi Y, Jun I, Tetsuro M, Hiroo F, Taku D (2005). Transcription switches for protoxylem and metaxylem vessel formation. Genes Dev.

[32] Ruiqin Z, Richardson EA, Zheng-Hua Y (2007). Two NAC domain transcription factors, SND1 and NST1, function redundantly in regulation of secondary wall synthesis in fibers of Arabidopsis. Planta.

[33] Mitsuda N, Iwase A, Yamamoto H, Yoshida M, Seki M, Shinozaki K, OhmeTakagi M (2007). NAC transcription factors, NST1 and NST3, are key regulators of the formation of secondary walls in Woody tissues of Arabidopsis. Plant Cell.

[34] Mitsuda N, Ohme-Takagi M (2010). NAC transcription factors NST1 and NST3 regulate pod shattering in a partially redundant manner by promoting secondary wall formation after the establishment of tissue identity. Plant J.

[35] Chengsong Z, Utku A, Grant EH, Haigler CH, Beers EP (2010). XND1, a member of the NAC domain family in Arabidopsis thaliana, negatively regulates lignocellulose synthesis and programmed cell death in xylem. Plant J.

[36] Qiao Z, Lina GG, Huanzhong W, Yining Z, Shi-You D, Fang C, Dixon RA (2010). An NAC transcription factor orchestrates multiple features of cell wall development in Medicago truncatula. Plant J.

[37] Zhang J, Huang GQ, Zou D, Yan JQ, Li Y, Hu S, Li XB. The cotton ( Gossypium hirsutum ) NAC transcription factor (FSN1) as a positive regulator participates in controlling secondary cell wall biosynthesis and modification of fibers. New Phytol. 2017;217. 10.1111/nph.14864 29105766

[38] Jin HM, Wei P (2011). Anti-fatigue properties of tartary buckwheat extracts in mice. Int J Mol Sci.

[39] Karki R, Park CH, Kim DW (2013). Extract of buckwheat sprouts scavenges oxidation and inhibits pro-inflammatory mediators in lipopolysaccharide-stimulated macrophages (RAW264.7). J Integr Med.

[40] Tsai H, Deng H, Tsai S, Hsu Y (2012). Bioactivity comparison of extracts from various parts of common and tartary buckwheats: evaluation of the antioxidant- and angiotensin-converting enzyme inhibitory activities. Chem Cent J.

[41] Hosoyama H, Mitomi A, Sagesaka Y, Kakuda T (2008). Endothelium-dependent vasorelaxation effect of rutin-free tartary buckwheat extract in isolated rat thoracic aorta. J Nutr Biochem.

[42] Nuruzzaman M, Manimekalai R, Sharoni AM, Satoh K, Kondoh H, Ooka H, Kikuchi S (2010). Genome-wide analysis of NAC transcription factor family in rice. Gene.

[43] Borrill P, Harrington SA, Uauy C (2017). Genome-Wide Sequence and Expression Analysis of the NAC Transcription Factor Family in Polyploid Wheat. G3 Genesgenetics.

[44] Le DT, Nishiyama R, Watanabe Y, Mochida K, Yamaguchishinozaki K, Shinozaki K, Tran LSP (2011). Genome-wide survey and expression analysis of the plant-specific NAC transcription factor family in soybean during development and dehydration stress. DNA Res.

[45] Shiriga K, Sharma R, Kumar K, Yadav SK, Hossain F, Thirunavukkarasu N (2014). Genome-wide identification and expression pattern of drought-responsive members of the NAC family in maize. Meta Gene.

[46] Singh AK, Sharma V, Pal AK, Acharya V, Ahuja PS (2013). Genome-wide organization and expression profiling of the NAC transcription factor family in potato (*Solanum tuberosum* L.). DNA Res.

[47] Hu W, Wei Y, Xia Z, Yan Y, Hou X, Zou M, Lu C, Wang W, Peng M (2015). Genome-wide identification and expression analysis of the NAC transcription factor family in cassava. PLoS One.

[48] Ma J, Wang F, Li MY, Jiang Q, Tan GF, Xiong AS (2014). Genome wide analysis of the NAC transcription factor family in Chinese cabbage to elucidate responses to temperature stress ☆. Sci Hortic.

[49] Diao W, Snyder JC, Wang S, Liu J, Pan B, Guo G, Ge W, Dawood M (2018). Genome-wide analyses of the NAC transcription factor gene family in pepper (Capsicum annuum L.): chromosome location, phylogeny, structure, expression patterns, cis-elements in the promoter, and interaction network. Int J Mol Sci.

[50] Wei S, Gao L, Zhang Y, Zhang F, Yang X, Huang D (2016). Genome-wide investigation of the NAC transcription factor familyin melon (Cucumis melo L.) and their expression analysis under salt stress. Plant Cell Rep.

[51] Su H, Zhang S, Yuan X, Chen C, Wang XF, Hao YJ (2013). Genome-wide analysis and identification of stress-responsive genes of the NAM–ATAF1,2–CUC2 transcription factor family in apple. Plant Physiol Biochem Ppb.

[52] Zhang L, Li X, Ma B, Gao Q, Du H, Han Y, Li Y, Cao Y, Qi M, Zhu Y (2017). The Tartary buckwheat genome provides insights into Rutin biosynthesis and abiotic stress tolerance. Mol Plant.

[53] Liu M, Ma Z, Wang A, Zheng T, Huang L, Sun W, Zhang Y, Jin W, Zhan J, Cai Y (2018). Genome-wide investigation of the auxin response factor gene family in Tartary buckwheat (Fagopyrum tataricum). Int J Mol Sci.

[54] Liu M, Fu Q, Ma Z, Sun W, Huang L, Wu Q, Tang Z, Bu T, Li C, Chen H (2019). Genome-wide investigation of the MADS gene family and dehulling genes in tartary buckwheat (Fagopyrum tataricum). Planta.

[55] Baranwal VK, Khurana P (2016). Genome-wide analysis, expression dynamics and varietal comparison of NAC gene family at various developmental stages in Morus notabilis. Mol Genet Genomics.

[56] Badouin H, Gouzy J, Grassa CJ, Murat F, Staton SE, Cottret L, Lelandais-Brière C, Owens GL, Carrère S, Mayjonade B (2017). The sunflower genome provides insights into oil metabolism, flowering and Asterid evolution. Nature.

[57] Shen Y, Liu J, Geng H, Zhang J, Liu Y, Zhang H, Xing S, Du J, Ma S, Tian Z (2018). De novo assembly of a Chinese soybean genome. Sci China Life Sci.

[58] Wang Y, van der Hoeven RS, Nielsen R, Mueller LA, Tanksley SD (2005). Characteristics of the tomato nuclear genome as determined by sequencing undermethylated EcoRI digested fragments. Theor Appl Genet.

[59] Dohm JC, Minoche AE, Daniela HW, Salvador CG, Falk Z, Hakim T, Oliver R, Thomas Rosleff SR, Ralf S, Richard R (2014). The genome of the recently domesticated crop plant sugar beet (Beta vulgaris). Nature.

[60] Jaillon O, Aury J-M, Noel B, Policriti A, Clepet C, Casagrande A, Choisne N, Aubourg S, Vitulo N, Jubin C (2007). The grapevine genome sequence suggests ancestral hexaploidization in major angiosperm phyla. Nature.

[61] Goff SA, Darrell R, Tien-Hung L, Gernot P, Ronglin W, Molly D, Jane G, Allen S, Paul O, Hemant V. A Recent Polyploidy Superimposed on Older Large-Scale Duplications in the Arabidopsis Genome[J]. Genome Res. 2003;13(2):137. 10.1101/gr.751803 PMC420368 12566392

[62] Korbinian S, Stephan O, Felix O, Klein JD, Xi W, Christa L, Smith LM, Joffrey F, Norman W (2011). Reference-guided assembly of four diverse Arabidopsis thaliana genomes. Proc Natl Acad Sci U S A.

[63] Vision TJ, Brown DG, Tanksley SD (2000). The origins of genomic duplications in Arabidopsis. Science.

[64] Blanc G, Hokamp K, Wolfe KH. **A** Recent Polyploidy Superimposed on Older Large-Scale Duplications in the. 2003;13. 10.1101/gr.751803 PMC420368 12566392

[65] Cannon SB, Mitra A, Baumgarten A, Young ND, May G (2004). The roles of segmental and tandem gene duplication in the evolution of large gene families in Arabidopsis thaliana. BMC Plant Biol.

[66] Prince VE, Bryan PF (2002). Splitting pairs: the diverging fates of duplicated genes. Nat Rev Genet.

[67] Cenci A, Guignon V, Roux N, Rouard M (2014). Genomic analysis of NAC transcription factors in banana (Musa acuminata) and definition of NAC orthologous groups for monocots and dicots. Plant Mol Biol.

[68] Liu L, White MJ, Macrae TH (2010). Transcription factors and their genes in higher plants functional domains, evolution and regulation. FEBS J.

[69] Hussey SG, Mizrachi E, Spokevicius AV, Bossinger G, Berger DK, Myburg AA (2011). SND2, a NAC transcription factor gene, regulates genes involved in secondary cell wall development in Arabidopsis fibres and increases fibre cell area in Eucalyptus. BMC Plant Biol.

[70] Duval M, Hsieh TF, Kim SY, Thomas TL (2002). Molecular characterization of AtNAM: a member of the Arabidopsis NAC domain superfamily. Plant Mol Biol.

[71] Krisztina N, Thomas B, Alexis P, Tetsuya I, Halima M, Mitsuhiro A, Patrick L (2006). The balance between the MIR164A and CUC2 genes controls leaf margin serration in Arabidopsis. Plant Cell.

[72] Ken-Ichiro H, Md Rezaul K, Shinobu T, Ken-Ichiro T, Masahiko F, Mitsuhiro A, Masao T (2006). Arabidopsis CUP-SHAPED COTYLEDON3 regulates postembryonic shoot meristem and organ boundary formation. Plant Cell.

[73] Agata B, Magdalena RS, Dorota BWT, Agnieszka P, Mitsuhiro A, Dorota K (2015). The CUP-SHAPED COTYLEDON2 and 3 genes have a post-meristematic effect on Arabidopsis thaliana phyllotaxis. Ann Bot.

[74] Liu M, Ma Z, Zheng T, Wang J, Huang L, Sun W, Zhang Y, Jin W, Zhan J, Cai Y (2018). The potential role of auxin and abscisic acid balance and FtARF2 in the final size determination of Tartary buckwheat fruit. Int J Mol Sci.

[75] Luo X, Zhao H, Yao P, Li Q, Huang Y, Li C, Chen H, Wu Q (2017). An R2R3-MYB transcription factor FtMYB15 involved in the synthesis of anthocyanin and Proanthocyanidins from Tartary buckwheat. J Plant Growth Regul.

[76] Liu M, Ma Z, Zheng T, Sun W, Zhang Y, Jin W, Zhan J, Cai Y, Tang Y, Wu Q (2018). Insights into the correlation between Physiological changes in and seed development of tartary buckwheat (*Fagopyrum tataricum* Gaertn.). Bmc Genomics.

[77] Ma N, Feng H, Xia M, Dong L, Yang D, Wu C, Meng Q (2014). Overexpression of tomato SlNAC1 transcription factor alters fruit pigmentation and softening. BMC Plant Biol.

[78] Chen M, Yang D, Ma X, Zhao W, Liang X, Ma N, Meng Q (2016). Suppression of tomato SlNAC1 transcription factor delays fruit ripening. J Plant Physiol.

[79] Selth LA, Dogra SC, Saif RM, Helen H, Randles JW, Ali RM (2005). A NAC domain protein interacts with tomato leaf curl virus replication accessory protein and enhances viral replication. Plant Cell.

[80] Huh SU, Lee SB, Kim HH, Paek KH (2012). ATAF2, a NAC transcription factor, binds to the promoter and regulates NIT2 gene expression involved in auxin biosynthesis. Mol Cells.

[81] Venkatesan S (2005). Control of seed size in plants. Proc Natl Acad Sci U S A.

[82] Weber H, Borisjuk L, Wobus U. Molecular Physiology of Legume Seed Development [J]. Annu Rev Plant Biol. 2005;56(1):253-79. 10.1146/annurev.arplant.56.032604.144201 15862096

[83] Mitsuda N, Seki M, Shinozaki K, Ohmetakagi M (2005). The NAC transcription factors NST1 and NST2 of Arabidopsis regulate secondary wall thickenings and are required for anther dehiscence. Plant Cell.

[84] Altschul SF, Madden TL, Schäffer AA, Zhang J, Zhang Z, Miller W, Lipman DJ (1997). Gapped BLAST and PSI-BLAST: a new generation of protein database search programs. Nucleic Acids Res.

[85] Finn RD, Jody C, Eddy SR (2011). HMMER web server: interactive sequence similarity searching. Nucleic Acids Res.

[86] Bateman A, Birney E, Durbin R, Eddy SR, Howe KL, Sonnhammer EL (2000). The Pfam protein families database. Nucleic Acids Res.

[87] Letunic I, Bork P (2018). 20 years of the SMART protein domain annotation resource. Nucleic Acids Res.

[88] Thompson JD, Gibson TJ, Higgins DG: Multiple sequence alignment using ClustalW and ClustalX. Curr Protoc Bioinforma 2002;Chapter 2(Unit 2):Unit 2.3. 10.1002/0471250953.bi0203s00 18792934

[89] Bailey TL, Mikael B, Buske FA, Martin F, Grant CE, Luca C, Jingyuan R, Li WW, Noble WS (2009). MEME SUITE: tools for motif discovery and searching. Nucleic Acids Res.

[90] Yupeng W, Haibao T, Debarry JD, Xu T, Jingping L, Xiyin W, Tae-Ho L, Huizhe J, Barry M, Hui G (2012). MCScanX: a toolkit for detection and evolutionary analysis of gene synteny and collinearity. Nucleic Acids Res.

[91] Livak KJ, Schmittgen TD (2001). Analysis of relative gene expression data using real-time quantitative PCR and the 2 −ΔΔ C T method. Methods.

